# Insights into treatment of complex Crohn's perianal fistulas

**DOI:** 10.1186/s12919-024-00291-4

**Published:** 2024-04-25

**Authors:** Gregor Norčič, Nataša Smrekar, Srđan Marković, Goran Barišić, Gediminas Kiudelis, Henrikas Paužas, Tamás Molnár, Attila Szijarto, Zuzana Šerclová, Tina Roblek, Viktor Uršič, Ian White

**Affiliations:** 1 University Medical Centre Ljubljana, Zaloška Cesta 2, Ljubljana, Slovenia; 2 Clinical Hospital Centre Zvezdara, Preševska 31, Belgrade, Serbia; 3 University Clinical Centre of Serbia, Pasterova 2, Belgrade, Serbia; 4 Lithuanian University of Health Sciences Kaunas Clinics, Eivenių G. 2, Kaunas, Lithuania; 5 University of Szeged, Dugonics Tér 13, Szeged, Hungary; 6 Semmelweis University, Üllői Út 26, Budapest, Hungary; 7 Military University Hospital, U Vojenské Nemocnice 1200, Prague, Czechia; 8 Takeda Pharmaceuticals, d.o.o., Bleiweisova Cesta 30, Ljubljana, Slovenia; 9 Beilinson Hospital, Rabin Medical Center, Ze’ev Jabotinsky Street 39, Petah Tikva, Israel; 10 Tel Aviv University, Ramat Aviv, Tel Aviv, Israel

**Keywords:** Crohn's Disease, Perianal Fistulas, Darvadstrocel Therapy, Multidisciplinary Management, Treatment Challenges, Surgical Procedures, Long-term Safety Evaluation

## Abstract

Complex perianal fistula is a common complication of Crohn’s disease (CD) which leads to negative impact on patient’s quality of life. Successful management of the disease requires a multidisciplinary approach, including a gastroenterologist and a colorectal surgeon, applying combined surgical and medical therapy. One of frequently practiced surgical procedures is seton placement in the fistula tract, which is used to control perianal sepsis and drain the fistula, while preventing recurrent abscess formation.

Darvadstrocel, a suspension of expanded, allogeneic, adipose-derived, mesenchymal stem cells, is safe and effective for treatment-refractory complex perianal fistulas in patients with Crohn’s disease. Following approval of darvadstrocel, the INSPIRE registry is being conducted in order to evaluate long-term safety and effectiveness of the drug on a large, heterogenous population.

An online expert meeting was held from March 20 to March 30, 2023, which provided relevant insights into the decision-making process regarding seton use and obtained feedback on the first experiences with darvadstrocel. The aim of this article is to present the perspectives from gastroenterologists and colorectal surgeons practicing in Czechia, Hungary, Israel, Lithuania, Serbia, and Slovenia in topics such as diagnosis and treatment options for patients with complex Crohn's perianal fistulas (CPF), specifically focusing on the use of setons and darvadstrocel.

During this virtual session, unavailability of comprehensive data on safety and efficacy of available treatment procedures was emphasized as an important obstacle towards development of standardized recommendations and improvement of outcomes in treatment of (CPF). Furthermore, achieving consensus in seton use, duration of its placement, and frequency of change is recognized as one of CPF treatments major challenges. Despite these issues, it is important to promote better understanding and treatment of complex perianal fistulas in order to improve the quality of life of those affected by this condition.

## Introduction

Perianal fistula is a frequent complication of Crohn’s disease (CD) which causes pain, discharge and inflammation in the perianal region resulting in significant impairment of patient’s quality of life [1, 2]. They arise from a pathological connection forming between the rectum or anus and surrounding tissues, including the perianal skin or other organs such as the vagina. [3]. Perianal fistulas are considered to be complex if they are not superficial, but rather high or above the sphincter complex (transsphincteric / extrasphincteric / high intersphincteric / suprasphincteric), if they are associated with multiple external openings, perianal abscess, and presence of rectovaginal fistula, anorectal stricture or active rectal disease at endoscopy [4]. Although various medications and surgical procedures exist, there is no standard treatment strategy. Successful management of the disease requires a multidisciplinary approach which includes gastroenterologists and colorectal surgeons, combining surgical and medical therapy [5]. Some of surgical techniques include seton insertion, fistulotomy, fistulectomy, fistula plug insertion, mesenchymal stem cell therapy, proctectomy with stoma creation etc., while most commonly used medical therapies are antibiotics, immunomodulators and biological therapy [1].

Setons are a common and effective technique to control perianal sepsis, drain the fistula and limit recurrent abscess formation prior to initiating medical treatment. They are placed in the fistula tract and may be left in situ for a longer period of time to control symptoms, whereas when the goal of treatment is fistula closure, setons must be removed [4, 6]. The ideal timing of removal remains matter of discussion.

Stem cell therapy represents one of novel treatment options with potential long-term healing. Darvadstrocel is a suspension of expanded adipose-derived mesenchymal stem cells which has been approved for the treatment of complex Crohn's perianal fistulas (CPF) in adult patients with non-active/mildly active luminal CD, when fistulas have not shown an adequate response to at least one conventional or biologic therapy [7]. Safety and efficacy of darvadstrocel have been evaluated based on the clinical trial known as ADMIRE-CD, or "Adipose derived mesenchymal stem cells for induction of remission in perianal fistulizing Crohn's disease". Results of this study indicated that darvadstrocel was well tolerated and clinical remission after the treatment and minimally invasive procedures, according to ADMIRE-CD study follow-up, may be sustained for up to 104 weeks [8]. Furthermore, the first real-world, multi-centre study evaluating effectiveness and safety of darvadstrocel for up to 36 months after the treatment in patients suffering from CPF is the INSPIRE registry. It is a post-approval, ongoing study, and the first published interim results were consistent with outcomes obtained from ADMIRE-CD study in terms of both safety and efficacy [9]. The INSPIRE study intends to include more patients in order to improve understanding of the disease, patterns of clinical care and outcomes in a large, heterogenous population [10].

The purpose of this report is to present the results from the Virtual Meeting which took place from March 20 to March 30, 2023 to provide relevant insights into the first experiences of using darvadstrocel in treatment of complex Crohn's perianal fistulas, as well as to identify main challenges in seton use. A total of 6 gastroenterologists and 4 colorectal surgeons from 5 European countries (Czechia, Hungary, Lithuania, Serbia, and Slovenia) and Israel participated in the meeting.

### Experiences with darvadstrocel treatment of complex perianal fistulas

All experts that took part in this event agreed that darvadstrocel has a favourable safety profile, while efficacy differs depending on severity of the disease and existence of comorbidities. In case patients do not respond to the treatment, some experts recommend re-evaluating the patient, determining the cause of therapy failure, and retreating the patient with darvadstrocel, or considering other therapy options.

Regarding evaluation of treatment response, all experts indicated that they always perform a clinical examination based on gentle finger pressure and evaluation of symptoms of the disease. Most of them stated that pelvic MRI is a more accurate diagnostic method than gentle finger pressure [11], but their healthcare institution’s funds allow them to perform it only in cases of deep abscess or suspected treatment failure. MRI is in those situations performed 6 – 12 months after administration of darvadstrocel [12]. In addition, experts from Hungary stated that they perform perineal ultrasound during every patient’s visit and a determination of Perianal Disease Activity Index (PDAI). PDAI is a scoring system which evaluates severity of perianal Crohn's disease based on 5 parameters: discharge, pain, restriction of sexual activity, type of perianal disease and degree of induration [13].

When discussing treatment effectiveness, it is important to define the precise meaning of responders and non-responders to therapeutic procedures. Experts that took part in this event defined responders to darvadstrocel treatment as patients who have no external secretions from fistulas, pain, or inflammation around the affected area and the fistulas are completely healed. If fistulas are not closed yet, but there are signs of healing and less secretions, then the patient is a partial responder to the treatment and no other surgical or medical procedures are required. In case a patient develops an abscess, and secretion and inflammation are not reduced, then the patient is not responding to the applied treatment, therefore it is necessary to consider other therapeutic options. One of the participating gastroenterologists pointed out that responding to treatment should be distinguished from remission. In the ADMIRE study, remission is defined as the closure of all treated external openings that were draining at baseline, coupled with the absence of collections larger than 2 cm of the treated perianal fistulas in at least two of three dimensions, confirmed through masked central MRI. Clinical assessment of closure entails the absence of draining despite gentle finger compression. Response, on the other hand, refers to the closure of at least 50% of all treated external openings that were draining at baseline by week 24 [8].

In his opinion, responder is a patient who subjectively feels better or symptoms of the disease have decreased, but his condition is not necessarily improved. Several participants stated that luminal recurrence of the disease, or a relapse of existing Crohn’s disease very early after the treatment, may contribute to relapse of CPF after treating with darvadstrocel.

One of the topics in this online event was ADMIRE CD study [8, 14], which, as far as participating experts are concerned, greatly reflects the real-world setting. They agreed that they mostly use the study’s protocol because patient profiles from the study resemble patients encountered in every-day practice.

When deciding whether a patient is eligible for darvadstrocel treatment or not, a few participants stated that a patient is eligible for the treatment if he/she has no or minor luminal symptoms of the disease (which is confirmed by colonoscopy) and on MRI scan the presence of no more than three external openings, associated collections, and a maximum of two internal and three external openings [7]. Finally, surgical examination confirms if the patient is suitable for the treatment or not. One of the experts added that patients with mild anorectal stenosis, deformation, scars in rectal mucosa, as well as patients who underwent advancement flap procedure which failed or a relapse occurred, are potential patients for administration of darvadstrocel. All participants stated that it is crucial to involve a multidisciplinary team in this decision-making process in order to achieve better treatment outcomes and higher rates of remission. In almost all cases, a colorectal surgeon and a gastroenterologist are included, but only an expert from Hungary indicated that a radiologist is also a part of their multidisciplinary team.

Most participating experts agreed that they have not experienced any challenges regarding logistical procedure and preparation of darvadstrocel, due to excellent organization and planning. Whereas, one of them pointed out a difficulty during transferring the content of the vial into the syringe which was solved by using an additional ventilation needle. The importance of having a back-up patient prepared, in case of acute illness of the patient for which the treatment was originally intended, was highlighted. Regarding administration of the drug itself, the majority of participants indicated that they had no problems with it, but some experts experienced issues while delivering the darvadstrocel solution to the affected area if the fistula is long and curved, and if extensive scarring around the fistula is present. Additionally, it was stated that when treating multiple, long fistulas, there is often not enough of the drug’s solution. As mentioned by a Hungarian surgeon, prolongation of financial reimbursement process of darvadstrocel within healthcare institution can cause delayed administration of the drug. In the meantime, patient’s disease might progress and the response to applied therapy may not be as expected. One of the participating gastroenterologists does not recommend combining darvadstrocel treatment and mucosal flap due to possibility of drug’s solution leakage.

According to participants in this online event, patients are fully involved in the decision-making process regarding therapy selection. The experts tend to inform patients about all treatment options, possible complications, and success rates of suggested treatments, but level of patient’s involvement often depends on their education and knowledge.

Regarding the use of MRI in seton treatment, most experts agreed that it is performed only when deciding on placement of seton, while seton removal is usually completed without using MRI [11]. In addition, relevance of this diagnostic method in cases of complications, multiple or combined fistulas, was highlighted. In experts’ opinion, MRI is not widely utilized due to its limitations, such as the high cost of the procedure, limited availability, and reliance on experts for interpretation, particularly in regions such as South East Europe where it is not always readily available.

INSPIRE registry is generally well accepted among colorectal surgeons and gastroenterologists that took part in this event. It was emphasized that the registry has the potential of providing real-world data regarding safety and effectiveness of darvadstrocel on a larger population [9, 10]. On the other hand, some participants mentioned that the registry would be very useful if it was not complex and if it would be made widely available. In support of that, a Serbian expert stated that Serbia is not included in mentioned registry due to only recent availability of darvadstrocel in their country. It was also suggested that other available surgical procedures could be included in the INSPIRE registry. When considering patient recruitment into this study, all participants agreed that they do not expect any challenges in identifying suitable patients, but rather in following up the patients according to the study protocol due to patient management issues on the site. Regarding collection of patients’ data and entering it into the e-platform of the study, the experts did not encounter any issues.

Identified key challenges in treatment of complex Crohn's perianal fistulas are early detection and monitoring of the disease, prevention of late, aggressive complications and preservation of patient’s quality of life. Additionally, it was pointed out by several experts that it is important not to jeopardize patient’s faecal continence while treating the fistula. In order to improve treatment outcomes and get clear, unambiguous recommendations in treating such a complex disease, it is necessary to perform additional clinical trials in this field, especially regarding combinations of surgical and medical therapy. Furthermore, it was stressed that patients with CPF should be treated only in highly-specialized centres, by experienced colorectal surgeons. One of the participants indicated that, concerning individualization of CPF therapy, it would be useful to develop predictive biomarkers that could improve risk assessment and foresee disease’s progress. Additional issues detected by the experts are concerning lack of long-term follow up data in patients suffering from CPF, insufficient data focusing on pathogenesis and treatment of complex perianal fistulizing Crohn’s disease and, regarding seton placement, a clear definition of a seton failure.

Comparing to MRI, the experts do not have a positive impression of VAAFT (Video-assisted anal fistula treatment) because, in their experience, it did not produce better results. Several participants emphasized the importance of MRI-based 3D reconstruction, which has the possibility of differentiating active disease from scarred tissue. For this reason, they do not see the benefit of incorporating VAAFT into future morphological fistula studies.

Even though darvadstrocel is approved by regulatory authorities in some countries, there are still several setbacks to its’ wider administration. For example, its’ cost and reimbursement issues have been recognized as an obstacle by almost all participating experts. Additionally, proper education of medical staff included in treatment of CPF could aid in better selection of patients who might benefit from darvadstrocel therapy.

In this online event it was emphasized the importance of communication between physicians and patients/patient associations in order to increase awareness about issues experienced by patients suffering from CPF. Beside physician–patient communication, correspondence among physicians, especially between experienced experts from IBD (Inflammatory Bowel Disease) centres and other specialists who encounter such patients, is also crucial for accomplishing optimal treatment of CPF. One of the participants pointed out the role of an IBD and surgical nurse, who as a part of the multidisciplinary team, often represents a bridge in communication between physicians and patients.

### Management and challenges in treatment of complex perianal fistulas with setons

One of treatment options that is frequently used in patients with complex perianal fistulas, is placement of a seton into the fistula tract. According to most of the experts who participated in this event, main long-term goals of seton placement are achieving mucosal healing, closure of the fistula without recurrence after seton removal, prevention of abscess formation, sepsis, and development of new tracts, and improving patient’s quality of life. On the other hand, one of the participating gastroenterologists suggested that placing setons, in his opinion, has only short-term goals, such as, drainage of fistula content and prevention of infections. Furthermore, he indicated that while a seton remains in the fistula, only epithelization of the tissue can occur, but it will unlikely result in complete healing of the fistula. Regarding methods used for evaluation of patient’s quality of life, one of the participants mentioned that she uses ProQOL (Professional Quality of Life Scale) [15], while some mentioned that they do not practice any official questionnaires, but assess it according to patient’s subjective feeling.

Viewing from patients’ perspective, participants estimated that the most relevant long-term goal in seton placement is improving quality of life, which includes minimizing symptoms of the disease, preventing faecal incontinence and complications, and enhancing the possibility of fistula’s permanent healing. It was also mentioned that inclusion of a psychologist into a multidisciplinary team could benefit patients’ understanding of their condition. Additionally, several participants stated that they often use drawings to aid in familiarizing patients with their disease, which is considered to be a relevant educational method. Level of patient’s knowledge and understanding of the disease is crucial for them to be included in the treatment decision-making process. Participants in this online event agreed that the majority of patients shows interest in being involved in their treatment selection and are mostly aware of all therapy options, while others rely on experts’ opinion. Patients tend to opt for the treatment that has a higher chance of success and is considered safer, rather than choosing surgical treatments or procedures that may lead to complications such as fecal incontinence or stoma formation, which they may not be comfortable with.

While discussing patients’ involvement in treatment decision-making process, attention was given to recently published expert consensus. It introduces a new classification system in which patients are allocated in groups depending on disease severity and outcomes, but simultaneously synchronizing patient’s and expert’s goals in decision making. This new classification system enables flexibility where patients can move between classes over time. Furthermore, there are suggested treatment strategies which is an important step towards standardization of clinical practice in the real-world setting [16]. According to experts that took part in this event, this new approach emphasizes the importance of patient’s expectations of treatment during decision-making process. As outlined in the consensus, it is necessary to intervene earlier, to commence with more invasive therapeutic procedures, while constantly reassessing patient’s goals of treatment. This approach primarily applies to patients in Classes 2, 3, and 4. Additionally, participants in this online event stated that aims of treatment, viewed from their perspective, mostly include complete fistula healing and prevention of future recurrence. They pointed out that often mentioned objectives are difficult to achieve, therefore improving patient’s quality of life is the most important treatment purpose.

Duration of setons remaining in fistula tracts varies greatly from patient to patient. When used in combination with medical therapy or surgical procedures, setons are kept for a shorter period of time, usually from 3 months, up to 1 year, as stated by experts who participated in this online event. If medical therapy fails, or it is necessary to prevent abscess and sepsis, setons can be left in the fistula long-term, for example, several years, or even, permanently. In general, clinicians’ goal is to remove the seton as soon as patient’s condition allows them to, i.e., when the symptoms decrease and the luminal disease is favourably controlled. In those cases, setons will be removed in order to improve patient’s quality of life. On the other hand, nonresponding to medical therapy, high risk of abscess formation and development of sepsis, generate a need for prolonging duration of seton placement in the fistula [5].

During long-term placement of setons, most experts indicated that, once the seton is placed, they do not regularly change it, unless it accidentally tears, falls out, or an additional fistula appears. Only a few of the participating experts stated that they change the seton yearly, even though no complications have occurred.

Several participants agreed that the duration of seton use inversely correlates to the chance of complete fistula healing. In their opinion, after a few months fibrotic changes occur, which prevent the healing, but these changes can be reversed by intense curettage of the fistula tract.

When treating or following up patients with complex perianal fistulas in Crohn’s disease, participants stated that almost all of them undergo a pelvic MRI. MRI is a diagnostic tool that distinguishes simple from complex perianal fistulas, allows precise definition of the fistula’s tract, and can distinguish between actively inflamed tracts vs fibrotic tracts, as well as identify abscesses. During deciding on treatment of fistulas, majority of participating experts agreed that the most precise result will be achieved by performing a combination of MRI and EUA (Examination Under Anaesthesia). Only several experts stated that they regularly perform Ultrasound, while MRI is solely used in cases of complex fistulas. The specificity and sensitivity of both imaging techniques is increased when combined with EUA [11]. Additionally, DRE (Digital Rectal Exam) was mentioned by one of the experts, as a method that, if possible, he routinely performs on his patients.

As mentioned before, approach to seton use among clinicians, varies greatly. The participating experts agreed that this disease is very complex and every patient should be assessed individually, therefore they do not consider it possible to develop uniform guidelines in seton procedures. Nevertheless, a few participants stated that they believe it would be possible to issue some recommendations, but only to a certain extent, as it would not be applicable to all patients. Generally, the seton placement itself is, more or less, similar among patients, but its’ removal, frequency of replacement, especially in complex cases, differs significantly. The participants believe that in specialized centres, existing recommendations are followed, whereas deviations occur in centres which do not have a multidisciplinary team consisting of educated and well-experienced staff. One of the experts mentioned that conduction of additional clinical trials offers a big step forward in improvement of standardization of clinical care in patients with CPF. Additionally, a Slovenian surgeon expressed his intentions in uniforming certain aspects of surgical treatment, for example, duration of seton placement, but also in promoting collaboration among specialized IBD centres on a national level.

Before applying darvadstrocel in patients with CPF, it is necessary to perform curettage in order to remove granulation tissue. Three out of five participating experts that provided answers regarding this topic indicated that they almost always perform two curettages of the same fistula before administration of darvadstrocel, usually with a period of 3–4 weeks between them. Exceptionally, only the first curettage is performed if there is no change in clinical picture after it. On the other hand, others stressed that only the curettage immediately before administration of darvadstrocel is essential due to possible logistical issues which could cause postponing of therapy administration, and in the meanwhile, reepithelization of the tract may occur. The surgical protocol for stem cell delivery that they mostly use is the one described in *Mesenchymal stem cells in perianal Crohn’s disease* by Guadalajara et al., where standardized key steps in this minimally invasive approach are outlined [17].

Lastly, the participating experts discussed characteristics of complex perianal fistulas in Crohn’s disease that they recognized as important factors in selecting patients who are most likely to respond to darvadstrocel therapy. Some of those factors include presence of no more than 3 fistula tracts, with length up to 5–6 cm. Additionally, patients who achieved complete mucosal healing after drainage of abscess and in who extreme fibrosis and epithelization of the tract have not occurred, could have a greater chance in benefiting from darvadstrocel therapy. Responders to mentioned treatment are also more likely to be patients who do not suffer from proctitis and have no or minimal luminal disease activity. According to the experts’ opinion, darvadstrocel could be used before biologics in patients who have complex perianal fistulizing Crohn’s disease, only if the disease is localized in the perianal region, with merely slight luminal activity.

## Conclusion

The Virtual Meeting delivered insights into the challenges of using darvadstrocel and potential data gaps in seton use based on views of gastroenterologists and surgeons from Czechia, Hungary, Israel, Lithuania, Serbia, and Slovenia. Darvadstrocel received positive feedback from the participating experts who experienced only minor logistical issues during administration of therapy, while its safety and efficacy is considered promising. Regarding seton procedure, identified challenges are achieving and following consensus in seton placement, duration, and frequency of change. Generally, the biggest recognized unmet need is lack of data on safety and efficacy of available treatment options, as well as reduced understanding of the disease itself, which limit the possibility of developing standardized guidelines and improving outcomes in treatment of complex perianal fistulas. Additionally, patient’s expectations and goals of treatment should be prioritized during treatment decision-making process and constantly reassessed over time.

## Data Availability

All the transcripts, insights and comments are available at Takeda Pharmaceuticals d.o.o. and by the corresponding author.

## Abbreviations

CD: Crohn's disease
CPF: Crohn's Perianal Fistulas
DRE: Digital Rectal Exam
EUA: Examination Under Anaesthesia
IBD: Inflammatory Bowel Disease
INSPIRE: Investigating Safety and Effectiveness of darvadstrocel for Treatment of Complex Perianal Fistulas in Crohn’s Disease
MRI: Magnetic Resonance Imaging
PDAI: Perianal Disease Activity Index
VAAFT: Video-assisted Anal Fistula Treatment
